# Investigating the association between stress, saliva and dental caries: a scoping review

**DOI:** 10.1186/s12903-018-0500-z

**Published:** 2018-03-13

**Authors:** Svetlana Tikhonova, Linda Booij, Violet D’Souza, Karla T. B. Crosara, Walter L. Siqueira, Elham Emami

**Affiliations:** 1 Faculty of Dentistry, McGill University, 2001 McGill College Avenue, Montreal, QC H3A 1G1 Canada; 2 Department of Psychology, Concordia University, 7141 Sherbrooke St. West, Montreal, QC H4B 1R6 Canada; 3 CHU Sainte-Justine & Université de Montreal, Montreal, Canada; 4 Faculty of Dentistry, Université de Montréal, C.P. 6128, succ. Centre-ville, Montreal, QC H3C 3J7 Canada; 5 Schulich School of Medicine & Dentistry, The University of Western Ontario, London, ON N6A 5C1 Canada

**Keywords:** Saliva, Dental caries, Psychological stress, Anxiety, Depression

## Abstract

**Background:**

This scoping review addressed the question ‘what do we know about stress-related changes in saliva and dental caries in general population?’

**Methods:**

The review was conducted using electronic searches via Embase, MEDLINE, PsycINFO, CINAHL and WoS. All published human studies with both observational and experimental designs were included. Two reviewers independently reviewed eligible articles and extracted the data. The studies’ quality was assessed using the Effective Public Health Practice Project Quality Assessment Tool.

**Results:**

Our search identified 232 reports, of which six were included in this review. All six studies were conducted in children and used salivary cortisol as stress marker. The studies varied by design, types of stressors, children’s caries experience, methods of saliva collection. Four studies reported a positive association between saliva cortisol levels and caries (*p* < 0.05) while the other two reported no association (*p* > 0.05). The quality of the included studies was weak to moderate.

**Conclusions:**

There is lack of evidence about an association between stress-related changes in saliva and caries. Well-designed longitudinal studies with rigorous measurement technics for stress, saliva and dental caries are necessary. This will help to generate new insights into the multifactorial etiology of caries and provide evidence for a rational method for its control.

## Background

Dental caries remains one of the most prevalent chronic diseases worldwide placing a significant burden on individuals and healthcare systems [1, 2]. Accordingly, in 2010, the Global Burden of Disease Study indicated that more than 2.4 billion people worldwide are affected by untreated dental caries [2]. Caries has negative impact on general health and quality of life of individuals [3]. Pain, decrease in masticatory performance, alteration of diet and nutrition, loss of working hours, as well as unaesthetic appearance and reduction in social activities are direct and indirect sequelae of caries disease [3, 4].

The high prevalence of dental caries in certain groups of the society in combination with limited effectiveness of the traditional education-based efforts to improve oral hygiene behaviors for caries prevention [5, 6] highlights the necessity to develop new strategies in caries control. In this regard, several research groups have emphasized the need for in-depth investigation of psychosocial and biological pathways through which social environment affects dental caries [7–9]. Some emerging evidence suggests that stress could have a potential role in caries disease [7, 10, 11]. The link between caries disease and stress can be explained via different pathways. Some of which include (but are not limited to) alterations in life style and unhealthy behaviors (e.g., excessive sugar intake, neglect of oral hygiene) [12–14], as well as through stress-induced changes in salivary composition and salivary flow rate [15, 16].

Stress can be defined as a real or interpreted threat to the physiological or psychological integrity of an individual that results in a cascade of physiological and/or behavioral responses of the body to maintain homeostasis [17, 18]. There is a widely-recognized theory of allostatic load which explains the effects of stress on the human body [18]. Under chronic exposure to stress conditions, a ‘wear and tear’ of the allostatic systems (central nervous system (CNS), the autonomic nervous system (ANS), the hypothalamus-pituitary-adrenal axis (HPA)) accumulate [18]. Over time, the ANS system and HPA axis becomes dysregulated. Excessive secretion of hormone cortisol will overstimulate the glucocorticoid receptors in the body, and will alter the function of certain neurotransmitters (e.g., adrenaline, noradrenalin, serotonin), which can affect the CNS, emotional and cognitive function as well as metabolic and immune systems [18, 19].

Saliva maintains the homeostasis of the oral cavity through various functions such as lubrication, buffering action, maintenance of tooth integrity and antimicrobial activity [20]. Furthermore, salivary proteins/peptides play an important role in the adherence of the oral micro-organisms to the tooth surface [15] and in maintaining the equilibrium between remineralization and demineralization processes [21]. The innervation and secretion of salivary glands are regulated by the ANS system, that in turn, affects salivary proteins concentration and salivary flow rate [22]. Under repeated chronic stress conditions, the ANS system functions and consequently, the salivary glands function can get altered, which may increase risk of dental caries [23, 24]. On the other hand, caries-related chronic pain and dental procedures can in turn be associated with the increase of chronic stress load [25, 26]. Salivary cortisol level has been recognized as a valid measure of active free cortisol and as a potential stress biomarker [27]. Many correlational studies showed a positive association of cortisol levels with chronic diseases such as periodontal diseases, diabetes, cardio-vascular diseases [28, 29] as well as with dental caries [30, 31]. Some experimental studies have shown an increase in cortisol concentration as well as in salivary total protein and secretory IgA after an exposure to experimental stress [23, 32, 33]. In addition, changes in salivary composition and microbial adherence have been shown after experimental stress conditions [15].

Summarizing the above-mentioned evidence, several changes in composition and saliva secretion can occur under stress conditions that in turn may have an association with dental caries. We conducted this scoping review to address the question ‘what do we know about stress-related changes in saliva and dental caries in general population?’ The study objectives were: 1. to map published literature concerning an association between saliva stress-related changes and dental caries; 2. to identify potential knowledge gaps in this area of research.

## Methods

### Electronic searches and eligibility criteria

The scoping review was guided by Arksey and O’Malley’s methodological framework (2005) as well as by other relevant literature sources focusing on enhancing scoping review methodology [34–36]. Based on preliminary broad search and consultation with an expert librarian, the following key words and MeSH terms were determined: dental caries, saliva, salivary proteins, stress, psychological, anxiety, depression. In order to identify the relevant studies, electronic searches were carried out via OVID in Embase, MEDLINE, PsycINFO (1960 to 2016 Sep week 1), CINAHL (1998 to 2016 Sep week 1) and WoS (1998 to 2016 Sep week 1). The search was complemented by reference tracking in identified articles and manual searchers in dental journals (Caries Research; Journal of Dental Research; Community Dentistry and Oral Epidemiology from 2011 to 2016 year). The following recourses were used for grey literature search: the TripDatabase; websites of American, Canadian and British Dental Assocoations; the abstracts of IADR meetings (2002–2016). An example of the search strategy in Medline is presented in Appendix 1.

**Fig. 1:**
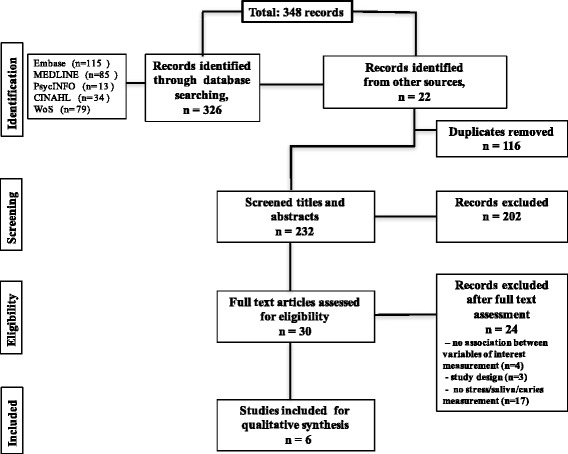
Diagram depicting process to search for and select final articles

Predefined inclusion criteria were: human studies with both observational (cohort, case-control, cross-sectional) and experimental (randomized clinical trial and quasi-experimental) designs investigating the association between stress-related changes in salivary composition/secretion (flow rate, proteins, salivary stress measures (e.g. cortisol) and dental caries). The search was restricted to articles written in English or French. Studies with insufficient data on salivary characteristics or dental caries, those that included patients with chronic diseases or conditions that can affect salivary function (e.g., Sjögren syndrome, rheumatoid arthritis, cancer), and /or taking medications such as antidepressants or glucocorticoids were excluded. Two reviewers (ST and VD) independently reviewed the titles and abstracts of the retrieved citations and identified eligible articles for full review. Inconsistency between reviewers was discussed with a third reviewer (EE) and resolved by consensus. All potential relevant studies were retained for full-text assessment (Fig. 1).

### Studies’ quality assessment

The Quality Assessment Tool for Quantitative Studies developed by the Effective Public Health Practice Project (EPHPP), Canada [37], was used to assess the quality of the included studies. This tool has demonstrated excellent inter-rater reliability as well as construct and content validity [38, 39]. The instrument included the following six components: sample selection, study design, confounders, blinding, data collection methods, withdrawals and dropouts. Each of these components was rated on a three-point Likert scale (strong, moderate and weak). A study was considered ‘strong’ if there were no weak ratings and with at least four strong ratings out of six. ‘Moderate’ were those with less than four strong ratings and one weak rating. Finally, ‘weak’ included those with two or more weak ratings. Quality assessment was performed independently by each reviewer (ST and VD), inconsistencies were resolved through discussion and with the research method expert (EE) if necessary.

### Data extraction and data analysis

Data was extracted using a pre-agreed data extraction form to gather relevant information from each selected study (e.g., authors, study design, study sample, measurement instruments for stress, saliva and caries, main findings), and the extracted data was charted. The charted data was summarized into a narrative synthesis.

## Results

### Study selection

Our search resulted in a total of 232 publications, of which 6 studies met the inclusion criteria and were included in the narrative synthesis. The selection process and the general characteristics of the selected studies are presented in Fig. 1 and Table 1 respectively.

**Table 1:** Selected studies for the systematic review

Publication	1. Rai K et al., 2010 India [44]
Study design	Quasi-experimental study (experimental group with 5 or more active caries lesions, history of pain; controls without caries history)
Study Sample	*n* = 60, children aged 5–10 years
Stressor/Stress measure	Dental treatment (e.g., oral prophylaxis, fluoride application, sealants, pulp therapy); done only for the experimental group Stress measure: saliva cortisol
Salivary parameters	Unstimulated whole saliva; Saliva cortisol collected before treatment and in 1, 2, 3 months after treatment; the time of the day was not indicated Measurement method: ELECSYS Immunoassay
Follow-up	No follow-up measurement for the control group
Caries measurement	DMFT and dmft index, WHO, 1997
Statistical test	Willcoxon signed rank test
Main results	Children with rampant caries had higher saliva cortisol level at the baseline then control group (*p* < 0.05). The baseline salivary cortisol level in children with rampant caries decreased gradually when observed for a period of three months following dental treatment.
Publication	2. Kambalimath et al., 2010 India [43]
Study design	Quasi-experimental study (experimental group with at least one carious lesion; controls without obvious caries)
Study Sample	*n* = 30, children aged 4–5 years
Stressor/Stress measure	Oral prophylaxis and topical fluoride treatment done for experimental and control groups Stress measure: saliva cortisol
Salivary parameters	Unstimulated whole saliva; Saliva cortisol collected before/after treatment (between 1 pm to 4 pm) at first and second appointments; Measurement method: Radioimmunoassay
Follow-up	Yes (first appointment post- treatment; recall appointment in one week)
Caries measurement	Not reported
Statistical test	T-test
Main results	No significant differences were found between the salivary cortisol levels prior to treatment, post oral prophylaxis, or post fluoride treatment at the first and second appointments of both groups (*p* > 0.05).
Publication	3. Yfanti et al., 2014 Greece [40]
Study design	Quasi-experimental study dmft≥3 – high degree of dental caries; dmft≤2 – low degree of dental caries
Study Sample	*n* = 97, children aged 6–10 years
Stressor/Stress measure	Dental treatment: a cleaning with rotary instruments or a small restorative procedure with the use of local anesthesia Stress measures: saliva cortisol; dental anxiety questionnaires
Salivary parameters	Stimulated whole saliva; Saliva cortisol collected before, after treatment (in 30 min) same day, same night, next morning, at recall Measurement method: Electrochemiluminescence Saliva alpha amylase collected before and after treatment, at recall. Measurement method: Enzymatic chromatometry
Follow-up	Yes (post-treatment, same night, next morning, recall visit in 7–14 days)
Caries measurement	DMFT (S), dmft (s), Koch criteria, 1970
Statistical test	Univariable linear regression
Main results	No significant associations were detected between cortisol and sAA levels and caries (*p* > 0.05).
Publication	4. Boyce et al., 2010 USA [30]
Study design	Cross-sectional study
Study Sample	*n* = 94, children ages 5–6 years from low SES families participating in a longitudinal study
Stressor/Stress measure	SES, family financial stress; Stress measure: saliva cortisol
Salivary parameters	Stimulated whole saliva; Salivary cortisol collected at first and last 20 min of the morning /evening school class, at the same time on each of three consecutive days. Measurement method: Immunoassay
Follow-up	No
Caries measurement	DMFS, WHO, 1997
Statistical test	Multivariate Poisson regression
Main results	Low SES, higher basal salivary cortisol secretion, and larger numbers of cariogenic bacteria associated with higher number of caries lesions (*p* < 0.001). The strongest risk factor for development of dental caries was the joint presence of heightened expression of salivary cortisol and high levels of cariogenic bacteria.
Publication	5. Barbosa et al., 2012 Brazil [31]
Study design	Cross-sectional study
Study Sample	*n* = 145, school children aged 8–14 years, low socio-economic status
Stressor/Stress measure	Stress measures: saliva cortisol; RCMAS (revised children’s manifest anxiety scale); CDI (children’s depression inventory)
Salivary parameters	Stimulated whole saliva; Diurnal decline (morning-night) in salivary cortisol Measurement method: Immunoassay
Follow-up	No
Caries measurement	dmft, DMFT, WHO, 1997
Statistical test	T test
Main results	Participants who experienced more dental caries had lower diurnal declines of salivary cortisol concentrations than participants with low caries experience (*p* < 0.05). No differences in anxiety and depression scores between individual with and without caries.
Publication	6. Pani et al., 2013 Saudi Arabia [45]
Study design	Cross-sectional study
Study Sample	*n* = 64, college-educated, working mothers aged 24–31 years *n* = 64 first born children aged 49–70 months
Stressor/Stress measure	Stress measure: salivary cortisol
Salivary parameters	Unstimulated whole saliva; Saliva cortisol collected two hours after waking up. Measurement method: Immunoassay
Follow-up	No
Caries measurement	DMFT (mothers) or dmft (children), WHO, 1997; bitewing radio-graphs
Statistical test	Mann-Whitney U test; Logistic regression
Main results	The mean salivary cortisol levels of children with ECC were significantly higher than caries free children (*p* < 0.001). The mean salivary cortisol levels of mothers of children with ECC were significantly higher than those of mothers of caries free children (*p* < 0.05). A significant correlation was determined between the salivary cortisol level of the mother and that of the child (*p* < 0.001).

DMFT(S) – decayed, missed and filed permanent teeth/surfaces because of caries; dmft (s) - decayed, missed and filed primary teeth/surfaces because of caries

### Characteristics of studies

The included studies were published between 2010 and 2014 and originated from United States, Brazil, Saudi Arabia, Greece and India. Among the six included studies, three were quasi-experimental and three cross-sectional. All six studies were conducted in children with the age range from 4 to 14 years. The sample sizes varied from 30 to 97 individuals in experimental studies and between 64 to 145 participants in observational studies.

The type of stressors varied across studies and they included: various types of dental treatment procedures (e.g., tooth cleaning, fluoride application, placing restorations) in quasi- experimental studies, while caries experience per se, dental pain, low socio-economic status and family financial stress were defined as chronic stressors in the included cross-sectional studies. The methods of saliva collection varied across studies: three studies used stimulated saliva and three studies used unstimulated saliva samples. All six studies used salivary cortisol as a stress marker. All studies used immunoassay system for measuring saliva cortisol. One study measured saliva protein alpha-amylase using enzymatic chromatometry [40]. None of the included studies reported on other salivary proteins. None of the included studies measured the salivary flow rate.

The DMFT(S) (decayed, missed and filed teeth/surfaces) index was used for recording of caries disease in five of the included studies: four studies applied WHO (World Health Organization), 1997 [41] caries diagnostic criteria, one study used diagnostic criteria of Koch, 1970 [42] and one study did not report on this issue.

#### Quasi-experimental studies

In all of three quasi-experimental studies (Table 1) the salivary cortisol level was measured in children with and without dental caries before and after dental treatment [40, 43, 44]. The baseline caries experience, number of saliva cortisol measurements per day, the specific time of the day, time and number of follow-ups (weeks/months) varied among the studies. In one study, a positive association between pre-treatment salivary cortisol level and caries was reported [44]. In addition, they also observed a steady decrease in the salivary cortisol level in children with rampant caries within three months after dental treatment [44]. Two other studies reported no association between salivary cortisol levels (pre-treatment/post-treatment/recall) and caries [40, 43]. In addition, no association between salivary alpha-amylase levels (pre-treatment/post-treatment/follow up) and caries was detected [40].

#### Observational studies

All three studies with observational designs (Table 1) were cross-sectional in nature [30, 31, 45]. The number of saliva cortisol measurements per day, and the time of the day varied among the studies. In all these studies, higher levels of salivary cortisol in children with caries disease were reported. One study showed that salivary cortisol levels of the mothers with children who had early childhood caries (ECC) were higher than the salivary cortisol levels of mothers who had children who were caries free [45].

#### Quality of reviewed studies

Quality assessment of the included studies is presented in Table 2. Based on the EPHPP Quality Assessment Tool [37], the global quality rating of the three included studies was moderate [30, 31, 40] and of the three remaining studies was weak [43–45]. Most of the studies were compromised with sample selection strategy and did not provide sufficient information on the validity and reliability of the measurement methods used, the confounding factors or adjusting for confounders in analyses.

**Table 2:** Studies quality assessment with EPHPP Quality Assessment Tool

Authors, year	Selection bias	Study design	Con-founders	Blinding	Data collection methods	Withdrawals and dropouts	EPHPP global quality rating
1. Rai K et al., 2010 [44]	Moderate	Moderate	Weak	Weak	Weak	n/a	Weak
2. Kambalimath et al., 2010 [43]	Moderate	Moderate	Moderate	Moderate	Weak	Weak	Weak
3. Yfanti et al., 2014 [40]	Moderate	Moderate	Moderate	Moderate	Moderate	Weak	Moderate
4. Boyce et al., 2010 [30]	Moderate	Weak	Moderate	Moderate	Moderate	n/a	Moderate
5. Barbosa et al., 2012 [31]	Moderate	Weak	Moderate	Moderate	Strong	n/a	Moderate
6. Pani et al., 2013 [45]	Weak	Weak	Moderate	Moderate	Moderate	n/a	Weak

Global quality rating: ‘strong’: no weak ratings and at least four strong ratings out of six; ‘moderate’: less than four strong ratings and one weak rating; ‘weak’: two or more weak ratings

*EPHPP* Effective Public Health Practice Project

## Discussion

In this scoping review, we systematically collected and examined the types and sources of scientific literature concerning the response of saliva to stress and its association with caries disease. This review focused on a broad range of possible stress-induced changes in salivary characteristics (e.g., changes in saliva flow rate, salivary proteins, immunoglobulins, cortisol, etc.), where only six studies measured saliva cortisol levels, as a measure of stress response. To control some confounders, studies with subjects who had chronic diseases/conditions (e.g., depression, cancer, etc.) and/or taking medications (e.g., antidepressants, corticosteroids, chemotherapy, radiation in the head and neck region) that may affect salivary function were excluded. Four out of six included studies (three cross-sectional and one quasi-experimental) found positive associations between saliva cortisol levels and caries while the other two studies reported no associations. Although this current review showed a possible positive association between salivary cortisol level as indicator of stress and dental caries, due to the small number of published literature and the methodological limitations of the included studies, our results do not permit to draw any firm conclusions. Yet, it identifies the knowledge gap and suggests that much remains to be done in this area of research.

According to the literature, numerous studies have reported changes in saliva composition and its properties after exposure to event-related stress [15, 23, 32, 33]. For instance, the increase in salivary protein concentration, as well as increase in secretory IgA concentration were found among young healthy adults (experimental stressors: public speech, laboratory exercise) [15, 23]. In addition, Bosh et al. [15] have reported that microbial colonization processes (adherence and co-adherence) were affected after event-related experimental stress, and these changes correlated with specific changes in salivary protein composition. Hugo et al. [16] have demonstrated that chronic psychological stress was associated with low stimulated saliva flow in adults. The absence of evidence on the aforementioned stress-induced changes in saliva and their association with caries may be explained by the following: 1. Dental caries is a multifactorial chronic disease and its causality investigation needs rigorous longitudinal study design, while the studies included in our review were quasi-experimental or cross-sectional in nature. 2. Most of the studies that revealed the changes in salivary composition were focusing on event-related stress and used experimental stressors. Thus, these studies were focusing on acute stress response while chronic response of saliva to stress may be different.

It is important to keep in mind a possible bi-directional association between stress and dental caries. Cohort study conducted in Dunedin, New Zealand has documented that dental fear in young adulthood was related to experience of high levels of dental caries and the tooth loss due to caries in mid- and late adolescence [46]. Thus, severe caries experience may be a co-adjuvant factor to chronic stress load.

### Strengths and weaknesses of the review

Many limitations should be kept in mind. Age, caries experience and saliva collection time were very variable in all the included studies. The methodological quality of included studies varied from weak to moderate. Most studies were compromised by study design, small study sample selection and sizes, measures and various methodological flaws (e.g., single point measurement of saliva cortisol, dental caries measurement criteria, blinding, non-random allocation, etc.). Despite the mentioned limitations, this scoping review was conducted systematically maintaining high quality in every step. Therefore, we could identify the existing knowledge gap in this area of research.

### Future recommendations for research

In view of the importance attributed to this topic and the identified knowledge gap, there is a high need to investigate the potential role of stress in caries disease through well designed and rigorous prospective cohort studies. One of the research focus may be related to the understanding of physiological mechanisms by which chronic stress exposures, related to low socio-economic status adversities, interact with biological body systems and consequently affect factors directly related to dental caries, such as saliva characteristics and tooth biofilm. When measuring stress, multiple methods are recommended focusing on 1. the sources of stress, 2. perception and the affective response to stressors and 3. the physiological stress responses. Each of the afforested approaches assesses different components of stress process [46]. Saliva cortisol has been acknowledged as a reliable indicator for HPA axis reactivity during the acute stress induction in experimental settings [24, 27]. However, the use of saliva cortisol as a chronic stress indicator has some limitations because of its secretion variability during chronic stress [47]. In addition, since cortisol secretion depends on circadian rhythm, multiple time point sampling during the same day and over time are necessary to completely capture stress-induced cortisol response [27]. Furthermore, several factors such as age, sex, menstrual cycle, drugs, diseases, time lag, and salivary flow rate could confound study results and should be considered [48, 49]. Since assessment of saliva cortisol as physiological indicator of stress is associated with several measurement complications which can affect the outcome, the measurement of hair cortisol level may be used as alternative method that represent the physiological response of the body to chronic stress [50].

## Conclusions

There is lack of evidence about an association between stress-related changes in saliva and caries. This study observed that more rigorous and analytical technics are needed for a precise measurement of saliva and tooth biofilm characteristics such as salivary proteome and oral biofilm microbiome analysis [51, 52]. Regarding the dental caries measurement methods, detailed caries diagnostic systems are recommended for use which consider severity and activity of caries lesions [53, 54]. In addition, a well-planned and rigorous cohort studies could provide better understanding of the role of stress in caries disease and would help generate new insights into the multifactorial etiology of dental caries. The combination of these approaches may provide strong evidence for a rational method of prevention/treatment of this worldwide disease.

## Appendix 1

### Search strategy example, MEDLINE

1. exp. Saliva/.

2. exp. Salivary Proteins/ and Peptides/.

3. “Salivary Proteins and Peptides”.nm.

4. saliva*.mp.

5. or/1–4.

6. exp. Dental Caries/.

7. exp. Dental Caries Susceptibility/.

8. caries*.mp.

9. white spot*.mp.

10. ((tooth or teeth) adj5 decay*).mp.

11. carious.mp.

12. or/6–11.

13. 5 and 12.

14. Stress, Physiological/.

15. exp. Stress, Psychological/.

16. exp. Anxiety/.

17. exp. Depression/.

18. stress*.tw.

19. (anxiet* or anxious*).tw.

20. depression.tw.

21. anguish*.tw.

22. or/14–21.

23. 13 and 22.

24. 5 and 13 and 22.

25. limit 24 to (yr = “1960 -Current” and (english or french)).

26. limit 25 to humans.

## Abbreviations

ANS: Autonomic nervous system
CHU: Center Hospitalier Universitaire
CIHR: Canadian Institutes of Health Research
CINAHL: Cumulative Index to Nursing and Allied Health Literature
CNS: Central nervous system
ECC: Early childhood caries
EMBASE: Excerpta Medica Database
EPHPP: Effective Public Health Practice Project
FRQS: Fonds de Recherche du Québec
HPA: Hypothalamus-pituitary-adrenal axis
IADR: International Association for Dental Research
IgA: Immunoglobulin A
IRSPUM: Institut de Recherche en Santé Publique de l’Université de Montréal
MEDLINE: Medical Literature Analysis and Retrieval System Online
MeSH: Medical Subject Headings
PsycINFO: Psychological Information Database
WHO: World Health Organization
WoS: Web of Science
